# Sialylated Oligosaccharides and Glycoconjugates of Human Milk. The Impact on Infant and Newborn Protection, Development and Well-Being

**DOI:** 10.3390/nu11020306

**Published:** 2019-02-01

**Authors:** Jolanta Lis-Kuberka, Magdalena Orczyk-Pawiłowicz

**Affiliations:** Department of Chemistry and Immunochemistry, Wrocław Medical University, Bujwida 44a, 50-345 Wrocław, Poland; jolanta.lis-kuberka@umed.wroc.pl

**Keywords:** human milk, nutrition, sialic acid, free oligosaccharides, glycoproteins

## Abstract

Human milk not only has nutritional value, but also provides a wide range of biologically active molecules, which are adapted to meet the needs of newborns and infants. Mother’s milk is a source of sialylated oligosaccharides and glycans that are attached to proteins and lipids, whose concentrations and composition are unique. Sialylated human milk glycoconjugates and oligosaccharides enrich the newborn immature immune system and are crucial for their proper development and well-being. Some of the milk sialylated oligosaccharide structures can locally exert biologically active effects in the newborn’s and infant’s gut. Sialylated molecules of human milk can be recognized and bound by sialic acid-dependent pathogens and inhibit their adhesion to the epithelial cells of newborns and infants. A small amount of intact sialylated oligosaccharides can be absorbed from the intestine and remain in the newborn’s circulation in concentrations high enough to modulate the immunological system at the cellular level and facilitate proper brain development during infancy. Conclusion: The review summarizes the current state of knowledge on sialylated human milk oligosaccharides and glycoconjugates, discusses the significance of sialylated structures of human milk in newborn protection and development, and presents the advantages of human milk over infant formula.

## 1. Introduction

Human milk is a highly complex fluid, which is produced locally by mammary epithelial cells, but also contains molecules that are filtered from the mother’s plasma. The biochemical analysis of human milk showed that the concentrations of individual components of mother’s milk are dependent on the gestational age [1,2], milk maturation stages [2,3,4,5], the health status of the mother [6,7], and perinatal risk factors [2]. Moreover, the composition of mother’s milk is related to the phase of the day [8], as well as the change during feeding (foremilk and hindmilk) [9]. Human milk, apart from nutrient balance, has an array of functional properties, namely anti-pathogenic, anti-infective, anti-inflammatory, and immunomodulatory. An important component of human milk is sialylated and fucosylated molecules, which constitute an essential element of innate immunity passed to the newborn with the mother’s milk. Currently, the WHO (World Health Organization) and ESPGHAN (European Society for Paediatric Gastroenterology Hepatology and Nutrition) guidelines on nutrition care for newborns and infants indicate that human milk is not only the best nutrient for the baby during the first six months of life but is also used as a medicine at neonatal intensive care units (NICUs) [10,11,12].

### Biological Role of Sialic Acid

**Figure 1:**
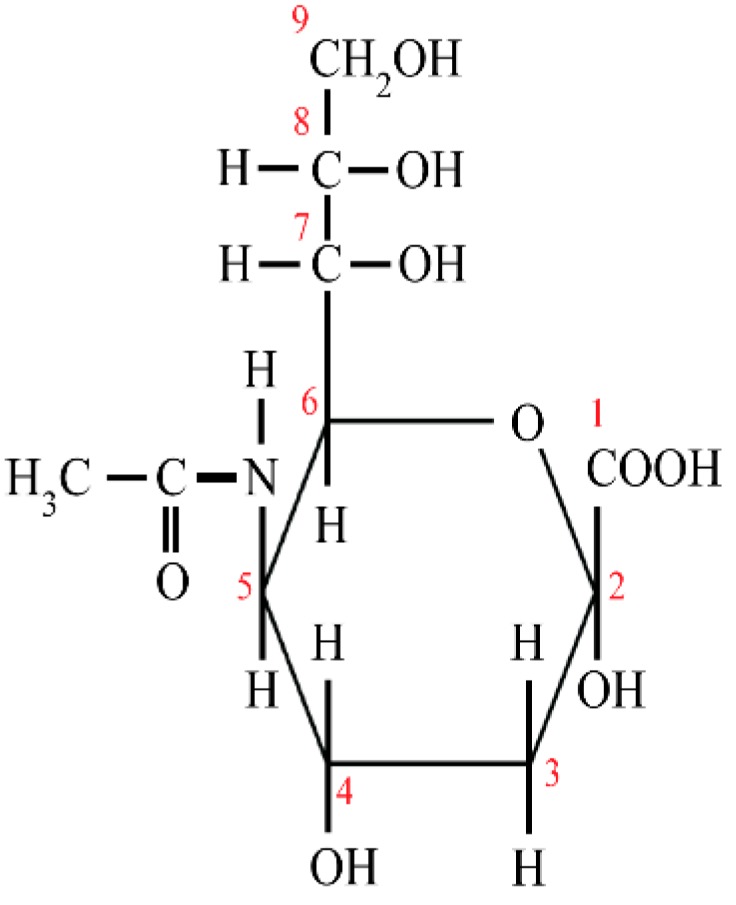
The structure of sialic acid. 1–9—the number of carbon atoms

N-acetylneuraminic acid (Neu5Ac), commonly known as sialic acid, is one of the more than 40 monosaccharides, and belongs to the family of derivatives of neuraminic acid (5-amino-3,5-dideoxy-D-glycero-D-galacto-non-2-ulosonic acid). Sialic acid is an acidic monosaccharide built up of nine carbon atoms, which, at anomeric carbon C2, has a free carboxyl group and at C5 an N-acetyl group (Figure 1) [13,14,15]. Sialic acid and fucose present in the structure at the terminal position of oligosaccharide chains according to the hypothesis of Gabius and coworkers [16] encode a significant amount of information, which is important in the regulation of signal transduction among cells and between cells and biomolecules. Such events are highly dependent on the composition, conformation, and sequence of monosaccharides, which form oligosaccharide chains/ and glycotopes [13,15,17]. Moreover, the presence of sialic acid in oligosaccharide chains of glycoproteins protect them against the removal from the circulation by specific asialoglycoprotein receptors of hepatic and Kupffer cells as well as by peritoneal macrophages [18].

Sialic acid may be linked by α2,3- and α2,6-glycosidic bonds, but the biological role of α2,3- and α2,6-linked sialic acids has been suggested to be quite different. Glycotopes containing α2,3-sialic acid, being a part of sialyl-Lewis x (sLeX) glycotopes, are classified as "carcino-embryonic" type, while containing α2,6-sialic acid as a "mature" type [13,15]. It was reported that the increase of α2,3- and/or α2,6-sialylated glycotopes on N- and/or O-glycans is associated with inflammation, cancerogenesis, and metastasis [19,20]. In addition, sialic acid might also form polysialic acid structures in which Neu5Ac molecules are connected to each other by α2,8 linkage [21,22]. Polysialic acid structures are critical for nervous system development and maintenance, and also for cancer metastasis, tissue regeneration, and repair and neuroinvasive potential of pathogenic bacterial strains [21].

Glycotopes containing sialic acid serve as a recognition determinant and can modulate many cellular mechanisms, proliferation of cells and differentiation of tissues [23,24], signal transduction [17], interactions between cells, cells and extracellular matrix, as well as cells and soluble ligands, thereby participating in biological recognition processes [14,15,17,25]. The participation of sialylated glycotopes in physiopathological processes, such as embryogenesis [26], fertilization [27], fetal development [24], selectin-mediated leukocyte adhesion to endothelium [28], apoptosis [29], inflammation [30], and cancer and immune diseases [15] has been confirmed. In addition, sialic acid plays an important role in the interactions between the host cells and pathogens, being a ligand for the lectin receptor of some sialic acid-dependent pathogens [15].

## 2. Sialylated Structures of Human Milk

Human milk is a rich source of sialylated structures such as human milk oligosaccharides (HMOs), glycoconjugates such as glycoproteins and glycolipids, and also contains sialic acid as a free molecule. The total sialic acid concentration in human term milk is the highest at the beginning of lactation and reaches 5.04 ± 0.21 mmol/L at days one–six, then decreases to the values of 1.98 ± 0.08 mmol/L and 1.04 ± 0.06 mmol/L at one and three months of lactation, respectively [17]. The most abundant sialylated human milk molecules are human milk oligosaccharides. About 70–83% of all sialic acid present in human milk is bound with HMOs, 14–28% with glycoprotein glycans and 0.2–0.4% with glycolipids (primarily gangliosides) [31,32]. The concentration and distribution of sialylated human milk glycoconjugates depend on milk maturation stages and the length of pregnancy [2,32,33]. The milk of mothers who delivered preterm contained about 13–23% more sialic acid compared to the term milk at the first and third months of lactation [32].

### 2.1. Sialic Acid in Human Milk Oligosaccharides

Thus far, more than 200 different structures of HMOs, built from 3–22 monosaccharides, have been identified [34,35,36,37,38]. However, based on the recent review by Urashima and coworkers, according to the Consortium for Functional Glycomics (CFG) of USA, 247 varieties of HMOs have been separated, of which 162 chemical structures have been characterized [39].

**Figure 2:**
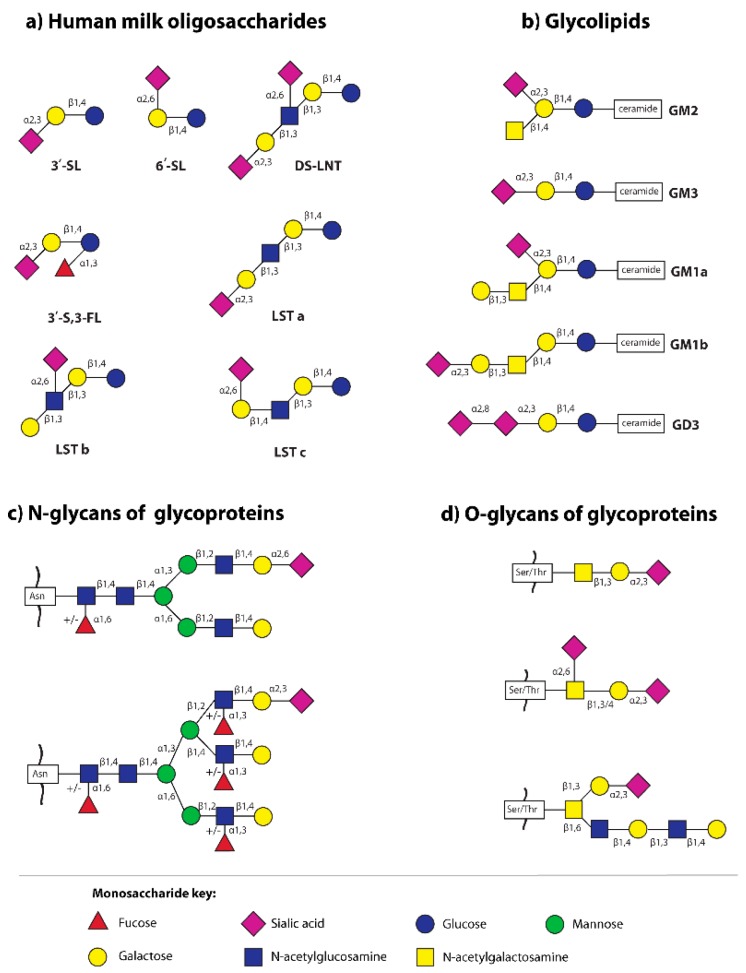
The main sialylated structures of human milk oligosaccharides and glycoconjugates (glycoproteins and glycolipids) [35,41,43,44]. (**a**) Human milk oligosaccharides: 3′-SL—3′-sialyllactose; 3′-S,3-FL—3′-sialyl-3-fucosyllactose; 6′-SL—6′-sialyllactose; DS-LNT—disialyllacto-N-tetraose; LST—sialyllacto-N-tetraose; (**b**) Glycolipids: GM—monosialoganglioside, GD—disialoganglioside; (**c**) Sialylated N-glycans of glycoproteins—the two most common structures in human milk N-glycome are presented [43]; Asn—asparagine; and (**d**) Sialylated O-glycans of human milk S-IgA [44]; Ser—serine, Thr—threonine.

The synthesis of HMOs takes place in the Golgi apparatus of maternal mammary alveolar cells [40]. For the majority of HMOs, the first step, namely the lactose core formation, is catalyzed by β4-galactosyltransferase one in the presence of α-lactalbumin [41]. In further steps, lactose can be elongated enzymatically by addition of monosaccharides such as N-acetylglucosamine (GlcNAc), galactose (Gal), fucose (Fuc), and sialic acid (Neu5Ac). The fucose can be attached by glycosidic linkage α1,2 to the galactose (Gal) and/or α1,3/4 to the GlcNAc, while sialic acid residues can be attached by α2,3 and/or α2,6 linkages to the terminal or subterminal galactose and/or N-acetylglucosamine (Figure 2) and cannot be substituted by other monosaccharides [41,42]. In a rodent model, β-galactoside sialyltransferases, namely ST3Gal4 and ST6Gal1 are involved in the biosynthesis of 3′- and 6′-sialyllactose, respectively [6], but data to prove their involvement in sialylated HMOs biosynthesis are missing [34]. Based on the presence of sialic acid, HMOs are divided into acidic oligosaccharides (with one or more sialic acid molecules) and neutral oligosaccharides without sialic acid molecules.

Due to structural complexity of HMOs, as well as lack of standards and different methods used by different laboratories for determinations of HMOs concentration, and as inter-individual differences among healthy lactating mothers, the available data can differ. The concentration of HMOs varies during milk maturation. The highest concentration is in colostrum 20 g/L and then substantially decreases to 16 g/L at day 30 of lactation [45]. The latest research by Xu and coworkers [46] showed that the concentration of HMOs at the 10th day of lactation was 19.3 ± 2.9 g/L and then decreased to the value 8.53 ± 1.18 at the 120th day of lactation. Moreover, the concentration of HMOs can vary depending on the week of delivery [47,48]. According to Morrow and coworkers [48], the concentration of total HMOs in preterm milk from days 10–23 of lactation was nearly two-fold lower than in term milk in the same period of lactation (3.6 g/L and 6.1 g/L, respectively). On the other hand, the latest research by Kunz and coworkers [49] showed that the total HMOs concentration in preterm milk did not differ significantly from term milk, either for colostrum (preterm: 8.7 g/L and term 7.5 g/L) or for transitional milk (preterm: 8.6 g/L and term: 9.1 g/L) or for mature milk (preterm: 8.6 g/L and term: 8.2 g/L). However, different mothers synthesize different subsets of milk oligosaccharides. The most extreme inter-individual variations are related to HMOs fucosylation and are based on mother’s secretor and Lewis blood group status. The majority of mothers express the α2-fucosyltransferase (FUT2), which is responsible for the addition of fucose via α1,2 linkage to terminal Gal [50] to form 2-fucosylated structures in milk (2’-fucosyllactose and LNFP-I are some of the most abundant) of so-called “secretor” mothers [51,52,53] in contrast to “non-secretor” mothers who do not express FUT2. The total HMOs concentration is “secretor” status-dependent [49,52]. In milk from “secretor” mothers, the total HMOs concentration was significantly higher compared with milk from “non-secretor” mother’s colostrum (median, 9.67g/L vs 5.17g/L), transitional (median, 9.47g/L vs 5.61g/L), and mature milk (median, 8.67g/L vs 5.54 g/L) [49]. 

The data concerning contribution of fucosylated, sialylated, and nonfucosylated neutral HMOs fractions are not unequivocal. According to Ninonuevo and coworkers [54,55], the proportions of fucosylated and sialylated HMOs in human term milk are 60–80% and 10–15%, respectively [54], and do not vary significantly at different stages of lactation [55]. On the other hand, as reviewed by Donnovan and Comstock [56], the content of fucosylated, sialylated, and nonfucosylated neutral HMOs in term milk is different, namely ~35–50%, 12–14%, and 42–55%. Moreover, as reported by Thurl and coworkers [52] and by Kunz and coworkers [49] milk samples of “secretor” and “non-secretor” mothers exhibited significant qualitative and quantitative differences in human milk oligosaccharides during the first three months of lactation. 

In contrast to fucosylated HMOs, the concentration of sialylated HMOs do not exhibit important differences among “secretor” mothers with Lewis blood group Le(a-b+) 2.23 g/l, “secretor” mothers with blood type Le(a-b-) 2.48, and for “non-secretor” mothers with Lewis blood group Le(a+b-) 2.17 g/l [49,52]. However, all milk groups exhibited an almost parallel decrease to one-third of the initial acidic oligosaccharides concentrations during the first three months of lactation [52]. Additionally, the concentration of sialylated HMOs in milk from secretor-mothers of healthy infants was significantly higher than in the milk of mothers of stunted infants [57].

The simplest sialylated oligosaccharides present in milk are trisaccharides, such as 3′-sialyllactose (3′-SL) and 6′-sialyllactose (6′-SL), which are formed by the addition of sialic acid by α2,3- and α2,6-glycosidic bonds to the galactose of lactose, respectively (Figure 2) [7,20,41,58,59]. The other oligosaccharides occurring in human milk containing α2,3- and/or α2,6-linked sialic acid are disialyllacto-N-tetraose (DS-LNT), and present in lower concentrations, sialyllacto-N-tetraoses (LSTa, LSTb, and LSTc) (Figure 2) [58].

Based on a summary published by Thurl and coworkers [58], the mean concentration of 3′-SL, 6′-SL and DS-LNT in term milk from secretor mothers during the first 100 days of lactation was 0.19, 0.64 and 0.50 g/L, respectively. Comparable data were reported by Donnovan and Comstock [56], namely for 3′-SL 0.2 (0.1–0.3) g/L and for 6′-SL 0.5 (0.2–1.22) g/L and by Sprenger and coworkers [60] at one month of lactation for 3′-SL 0.22 g/L and for 6′-SL 0.5g/L. Similar concentrations of 3′-SL and 6′-SL in preterm milk (0.24 and 0.60 g/L) during the first 30 days of lactation were observed [58]. Moreover, the concentrations of sialylated HMOs, namely 6′-SL, 3′-SL, and DS-LNT, as suggested by McGuire and coworkers [61], vary geographically. However, the authors pointed out that to confirm such as hypothesis targeted genomic analyses are required to determine whether these differences are due at least in part to genetic variation. The level of all sialylated HMOs was more than two times higher in the milk of mothers in Ghana (mean 3.6 mmol/L) than in milk of mothers in Sweden (mean 1.6 mmol/L). However, for two sialylated structures of HMOs, namely for 6′-SL and DS-LNT, the concentrations were more than four and 2.5 times higher, respectively, in milk of mothers in Ghana than in the milk of Swedish mothers [61].

### 2.2. Sialic Acid in Human Milk Glycoproteins

Sialic acid can be linked by α2,3- and/or α2,6-glycosidic linkages to the terminal galactose (Gal) of N-glycans and by α2,3- and/or α2,6-glycosidic linkages to the terminal galactose (Gal) and/or subterminal N-acetylgalactosamine (GalNAc) of O-glycans of glycoproteins (Figure 2) [41,43,44]. Nevertheless, the sialic acid can be substituted by other sialic acids linked by α2,8-glycosidic bonds to make polysialic acid, e.g., CD36 protein of the human milk fat globule outer membrane is decorated by O-glycans with polysialic acid [21,22].

57% of N-glycans of human milk glycoproteins are sialylated [43]. Additionally, the analysis of the sialylation profiles of secretory immunoglobulin A (S-IgA) and lactoferrin (LF) in milk of mothers with gestational diabetes mellitus showed a lower for S-IgA and higher for LF content of sialylated N-glycans compared to the milk of healthy mothers [7].

Detailed studies have shown that, during milk maturation, the sialylation pattern of human skim milk glycoprotein differs in relation to the type of sialic acid linkage to the glycan part of glycoprotein, as well as to the analyzed glycoprotein [4,5,33]. The overall relative amounts of α2,6- and α2,3-sialylated glycotopes of human milk glycoproteins at days 39–47 of lactation were 33% and 89% of those observed for colostrum, respectively [33]. However, for some glycoproteins no significant differences were observed during lactation. The overall changes observed for α2,6- and α2,3-sialylation patterns of human skim milk glycoproteins correspond to term milk maturation and overlap with trends for sialylated HMOs, namely, 6′-SL, 3′-SL, and DS-LNT [58] over lactation.

It is also worth paying attention to the fact that some glycoproteins, which were detected in human milk, are also found in plasma, although their glycosylation profile is quite different. The alternations in glycosylation patterns are related to some locally synthesized glycoproteins, namely immunoglobulin G (IgG) [2], α_1_-acid glycoprotein (AGP) [4], and fibronectin (FN) [5] by alveolar cells of the mammary gland, which is hormonally regulated [34,62].

### 2.3. Sialic Acid in Human Milk Glycolipids

In milk, glycolipids are found almost exclusively as a component of the outer layer of the milk fat globule membrane [9,63,64,65,66]. Glycolipids of human milk are divided into neutral glycolipids (glycolipids without sialic acid) such as glucosylceramide, galactosylceramide, and acidic glycolipids, with sialic acid in their structures, namely gangliosides, which are the most abundant fraction of human milk glycolipids [64,65,66,67].

The data concerning the total concentration of lipid-bound sialic acid (T-LBSA) during milk maturation are inconsistent. As reported by Pan and Izumi [67], the concentration of T-LBSA is not dependent on milk maturation stages, reaching 9.5 ± 1.2 mg T-LBSA/L in colostrum and 9.1 ± 1.2 mg T-LBSA/L in mature milk. On the other hand, Giuffrida and coworkers [68], using liquid chromatography coupled with electrospray ionization high resolution mass spectrometer (LC/ESI-HR-MS), reported a slight increase in total gangliosides from 8.1 mg/L during the first 11 days of lactation to the value of 10.7 mg/L at the 4th month of lactation. Moreover, differences in the content of particular fractions of gangliosides are also observed. The main gangliosides of human milk are monosialoganglioside 3 (GM3, Neu5Acα2,3Galβ1,4Glcβ1,1 ceramide) and disialoganglioside 3 (GD3, Neu5Acα2,8Neu5Acα2,3Galβ1,4Glcβ1,1 ceramide), while GM2 and GM1 represent a small fraction (2% and 0.1%, respectively) (Figure 2) [41,65,69,70]. It was reported that the concentration of human milk GM3 increases, while the concentration of GD3 decreases during lactation [69]. According to Giuffrida and coworkers [68], the concentrations of GD3 and GM3 in human milk at the beginning of lactation, between postpartum days zero and 11, were at a similar level (3.8 and 4.3 mg/L, respectively), while at day 30, the concentrations were 1.7 and 7.4 mg/L, respectively. On subsequent days of lactation the differences between concentrations of GD3 and GM3 were even greater, 0.9 and 9.1 mg/L, respectively, and then at day 120, remained at a stable level (0.9 and 9.8 mg/L, respectively) [68].

## 3. Metabolism of Sialic Acid by Infants

Karim and Wang [71], based on analysis of the enzymes involved in the synthesis of sialic acid in a rodent model, suggest that liver and other organs of newborns may not have the full ability to synthesize sialic acid during the first two weeks of their postnatal life. In light of the above, a very important part of the diet of newborns and infants is sialic acid delivered with mother’s milk. The high level of sialic acid in mother’s milk at the beginning of lactation is an evolutionary adaptation, to compensate for the partial capacity of the newborn and infant’s liver to synthesize endogenous sialic acid [71]. However, it should be pointed out that there are only speculations based on data from animal studies, which do not reflect the glycosylation pathways utilized by humans.

As sialic acid is delivered with mother’s milk, mainly in bound form (HMOs, glycoproteins and glycolipids), its metabolism in the body of newborns and infants involves a series of reactions to release this monosaccharide from glycoconjugates. In a rat model, high activity of neuraminidase in the intestinal mucosa is probably related to a high level of sialylated molecules in the milk [71]. The administration of guinea pigs with radiolabeled sialyllactose resulted in its ~90% absorption from the intestine within four hours, and then it was used as a substrate for the synthesis of new sialylated glycoconjugates of tissues (30%) and brain (~3%) [72]. On the other hand, intravenous administration of three-day old piglets with labeled free sialic acid showed that, within two hours, 0.23% was located in the brain [59,73].

In the plasma of partially breastfed infants the pattern of sialylated HMOs such as 3′-SL (~23%), 6′-SL (~3%), 3′-SLN (~14%), and 6′-SLN (~23%) was quantitatively different from exclusively formula-fed infants: 3′-SL (~51%), 6′-SL (~1%), 3′-SLN (~8%), and 6′-SLN (~10%), respectively [74]. These findings support the hypothesis that HMOs-related sialic acids are transported to the lumen of the small intestine or colon of newborns and infants and are then absorbed into their plasma [74]. Moreover, HMOs present in the bloodstream of infants are excreted with the urine. Additionally, a significantly higher level of 6′-SL (26.1 ± 17.2 mg/L) in urine of breastfed infants in comparison to formula-fed infants (0.9 ± 0.3 mg/L) was observed [75].

Surprisingly, in the urine and feces of both breast and formula-fed infants, the presence of 6′-sialyllactosamine (6′-SLN), which was not found in mother’s milk and plasma, was detected [75,76]. Simultaneously, Ruhaak and coworkers [74] confirmed the presence of 6′-SLN and a new, unknown isomer of SLN in infant plasma. The ‘modified’ HMOs in the newborn’s urine may be synthesized endogenously from smaller precursors [77]. Additionally, the “new” HMOs may be the result of bacterial fermentation of milk glycoconjugates or a part of glycan structures present on cell surfaces [78].

The metabolism and functions of HMOs are intensively studied based on an animal model. Jantscher-Krenn and coworkers [79] reported that, in a rat model, only 3′-sialyllactose and hardly any other HMOs appeared in the serum and the urine of HMOs-fed rats and suggested a selective absorption of rat milk-specific oligosaccharides. However, subsequent studies have shown that an oral administration of an individual HMO, such as 2′-FL, 6′-SL, and LNnT, which were partially absorbed into the plasma and excreted in urine impacted the concentration of other HMOs in serum and in urine of pups and adult rats. Additionally, such an effect was more evident when the higher doses of oligosaccharides were administered. Moreover, the observed changes were more detectable in urine than in serum. However, further studies are needed to understand this phenomenon [80].

## 4. Metabolism of Sialic Acid by Infants Microbiota

Microorganisms, which form the intestinal flora of newborns and infants, namely the microbiota, participate in the metabolism of human milk glycoconjugates [81]. Microbiota composition depends on the feeding type [82,83]. The intestinal microflora of breast-fed infants is dominated by *Bifidobacterium* strains (*B. breve*, *B. adolescentis*, *B. longum*, and *B. bifidum*) (approximately 73% of all bacteria), while *Bifidobacterium* (approximately 31%), *Bacteroides*, and *Enterobacteria* are typical for formula-fed infants [82,84]. Moreover, the differences in total bacteria, *Enterobacteriaceae*, and fecal ammonia among formula-fed infants with and without colic was reported [84].

The *Bifidobacterium* and *Lactobacillus* spp. present in the gastrointestinal tract of newborns and infants differ in their ability to utilize HMOs. *Bifidobacterium infantis* in comparison to *Lactobacillus gasseri* has a great ability to digest HMOs [85]. The genome of *B. infantis* encoded 24 glycosidases (including 2 α-sialidases and 5 α-L-fucosidases) [86]. Moreover, *Bifidobacterium bifidum* can release monosaccharides from HMOs, but has no ability to utilize fucose, sialic acid, and N-acetylglucosamine [87]. In contrast, *Bifidobacterium breve* cannot cut off monosaccharides from HMOs, but can ferment them [85,88]. Schwab and Gänzle [88] analyzed the hydrolytic activity of six strains of lactic acid bacteria—*Lactobacillus acidophilus*, *Lactobacillus plantarum*, *Lactobacillus fermentum*, *Lactobacillus reuteri*, *Streptococcus thermophilus*, and *Leuconostoc mesenteroides* subsp. *cremoris*—and reported that only two strains, namely *L. plantarum* and *L. acidophilus*, are able to hydrolyze sialylated HMOs, albeit with different efficiency. The hydrolytic activity of *L. acidophilus* and *L. plantarum* was observed for 3′-SL and 6′-SL, and additionally for 2′-FL, 3-FL and lacto-*N*-tetraose [88]. Moreover, glycosidases produced by commensal bacteria in the infant gut can in vivo produce a new, so far unknown SLN oligosaccharide structure [74].

## 5. The Significance of Sialylated Structures of Human Milk

### 5.1. Sialylated Glycans of Human Milk and Psychomotor Development of Newborns and Infants

Mammalian nerve cells, compared to other cells, exhibit a high content of sialic acid as a key component of gangliosides and neural cell adhesion molecules important during neurodevelopment. The majority of sialic acid present in the brain is associated with gangliosides (65%) and glycoproteins (32%), while free sialic acid constitutes less than 3% [89,90]. The amount and distribution of sialic acid are variable during pre and postnatal brain development [91,92]. The level of GM1 and GD1a gangliosides in the human frontal lobe of the brain showed an approximately 12-15-fold increase from the 10th gestational week to the age of about five years [91]. The analysis of distribution of a radiolabeled sialic acid in the rat brain showed that 80% was located in the vicinity of synapses, and can influence the functioning of membranes in the synaptic space [93]. Later studies [59,90,92] showed that sialic acid is involved in the transmission of signals between nerve cells and enhances cognitive function such as learning performance and memory. Human milk, especially at the beginning of lactation, is rich in sialylated structures, which might be an exogenous source of sialic acid used by newborns and infants for synthesis of new sialylated molecules [32,59,94]. Ruhaak and coworkers [74] speculate that small amounts of sialic acids or HMOs-related sialic acids present in the lumen of the small intestine or colon might be absorbed into the bloodstream, across the blood-brain barrier, reaching the immature brain.

In an animal model, the impact of orally administration of free and conjugated sialic acid on learning and memory is intensively studied [92]. In neonatal pigs, dietary supplementation of formula with 3’- or 6’-sialyllactose serves as a source of sialic acid for neurologic development and enrich ganglioside-bound sialic acid in the brain, however did not affect feed intake, growth, or fecal consistency [95]. Additionally, the potential effect of dietary sialyllactose on neurodevelopment in a newborn piglet model depends on the suplementation doses. Moreover, different parts of the brain, namely corpus callosum, prefrontal cortex, and hippocampus may be differentially sensitive to dietary sialyllactose administration [89]. The learning behavior of adult rats is improved by feeding of sialyllactose. Similarly to pigs, the brain ganglioside and GM3 contents were significantly higher after suplementation of rats with sialyllactose [96]. Recent studies have shown that providing of sialic acid during lactation in rats, enhanced long-term potentiation compared to controls. Moreover, some cognitive outcomes showed better scores for 6′-sialyllactose supplemented rats compared to free sialic acid supplemented rats [97].

### 5.2. Sialylated Glycans of Human Milk and Pathogen Adhesion

**Figure 3:**
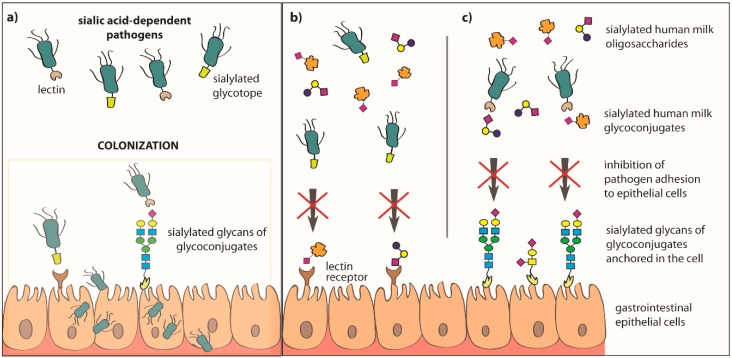
Inhibition of pathogen adhesion to epithelial cells of the newborn’s and infant’s gastrointestinal tract by sialylated oligosaccharides and glycoconjugates of human milk. The possibility of colonization of host epithelial cells by pathogens is called an invasive virulence mechanism, and in this process an important role is played by surface structures of microorganisms, such as lectin receptors and/or glycotopes (**a**). The process of adhesion of microorganisms to the host lectin receptor and/or glycoconjugates can be inhibited by the sialylated human milk oligosaccharides and soluble glycoconjugates, which can be recognized and bound by the lectin receptors of epithelial cells (**b**), and/or are recognized and bound by bacterial lectin receptors (**c**). In both cases, the occurrence of this type of interaction between sialylated oligosaccharides or glycoconjugates of human milk and bacterial lectin receptors and/or host cells leads to the inhibition of pathogen adhesion.

Among the many benefits of breastfeeding, one of the major advantages is the opportunity to significantly reduce the risk of intestinal diseases, as well as ear and respiratory tract infections in infants [34,98,99]. Human milk oligosaccharides and glycoconjugates, namely glycoproteins and glycolipids [70,94,100,101], are involved in the inhibition of pathogen adhesion to the host epithelial cells (Figure 3, Table 1) and they may act synergistically [66].

Soluble sialylated human milk oligosaccharides and glycoconjugates "rinsing" epithelial cells of the throat, esophagus, and intestines of the breastfed newborn can be recognized and bound by lectin receptors of sialic acid-dependent bacteria and/or lectin receptors of the host cells. In both cases, it leads to blocking of lectin receptors by sialylated milk molecules, which does not allow colonization of the host cells by pathogens (Figure 3) [10,34,66,118,119,120]. The participation of sialylated and fucosylated human milk oligosaccharides and glycoconjugates in the inhibition of pathogen adhesion to host cells is possible due to the presence on their structures of glycotopes, which are the same as or are very similar to the glycotopes of glycoconjugates present on the surface of host epithelial cells [66].

Human milk oligosaccharides elicit antimicrobial and antibiofilm activity against *Streptococcus agalactiae* (GBS), *Staphylococcus aureus*, and *Acinetobacter baumanni*, which are pathogens of particular interest in infant health [121,122,123,124]. As reported by Lin and coworkers [124] only neutral, non-sialylated fraction of HMOs may inhibit growth of group B *Streptococcus*. However, further investigation demonstrated that the five structurally defined, ubiquitous sialylated HMOs, variants of lacto-N-tetraose, exhibit antimicrobial and antibiofilm activities against Group B *Streptococcus* [125]. Moreover, HMOs have ability to potentiate the antibiotic activity what seem to be important as GBS has evolved high levels of resistance toward aminoglycosides, macrolides, and tetracyclines [126].

It was shown that 3′-SL and 2′-FL may in vitro reduce the incidence of viral infections caused by respiratory syncytial virus (RSV) by a significant decrease of RSV viral load and cytokine level in airway epithelia [99]. A similar effect was also observed for 6′-SL and LNnT for influenza viral load [99]. It has been reported that HMOs contribute to the reduced duration of rotavirus-induced diarrhea in a large animal model. Preclinical study in pigs showed that the dietary HMOs such as 2′-fucosyllactose, lacto-N-neotetraose, 6′-sialyllactose, and 3′-sialyllactose were more effective than prebiotics in altering systemic and gastrointestinal immune cells and may influence on rotavirus infection susceptibility [127]. Additionally, sialylated milk oligosaccharides can reduce the infectivity of human rotaviruses in monkey kidney epithelial cells (MA104), primarily through an effect on the virus [128]. Moreover, the mixture of 3′-SL and 6′-SL, at the same ratio as in breast milk, was more effective in reducing infectivity (73% reduction) than when compared with 3′-SL (47% reduction) or 6′-SL (40% reduction) individually [128].

Specific interaction between sialylated glycans of S-IgA and S-fimbriated *E. coli* protects newborns from sepsis and meningitis caused by these pathogens [108]. Additionally, human milk S-IgA glycans are an important element that links innate and acquired immunity [44]. Moreover, sialylated glycans of human milk κ-casein inhibited the binding of *Streptococcus mutans* GS-5 to saliva-coated hydroxyapatite [110], while sialylated glycans of milk mucins can be bound by rotavirus and inhibit its replication both in vitro and in vivo [111] (Table 1). It was also reported that Neu5Acα2,3Gal and Neu5Acα2,6Gal purified from human milk might inhibit the adhesion of enterovirus 71 to the human cell line DLD-1 [117]. Interestingly, some viruses such as coxsackie virus 24 bind preferentially to α2,3-sialylated glycans, in contrast to preferential binding of α2,6-sialylated glycans by influenza virus [129,130].

The human milk fat globule membrane contains gangliosides, which also participate in protection of breastfed newborns and infants against pathogens (Table 1). However, their efficiency is different, namely GM1 showed 80% inhibition of adhesion of enterotoxigenic strain of *E. coli* to the cell line Caco-2 (in vitro Caco-2 cell monolayer form functionally and structurally similar to human enterocytes), while GM3 and GD3 showed 69% and 16% inhibition, respectively [112]. Additionally, some sialylated glycolipids of human milk may also prevent adverse effects of cholera toxin [131], Shiga toxin [65], and heat-labile enterotoxin of *Escherichia coli* [70,132,133] (Table 1). Moreover, GM1, GM3, and GD3 glycolipids of human milk are able to reduce the adhesion of *Campylobacter jejuni*, *Helicobacter pylori*, *Listeria monocytogenes*, *Salmonella enterica* serovar Typhi, and *Shigella sonnei* to Caco-2 cells [70,116]. In light of the above, the different types of oligosaccharides and glycans attached to glycoconjugates present in human milk seem to cooperate to provide broader protection of newborns and infants against infections [64]. Moreover, they are considered as natural prophylactic or therapeutic biomolecules, which modulate and support the immature immune system of newborns and infants.

### 5.3. Sialylated HMOs and Altered Glycan-Related Gene Expression

Sialylated oligosaccharides of human milk may also modulate the glycosylation pattern of the surface of host (newborn and infants) epithelial cells. In vitro studies showed that the addition of 3′-SL decreases the expression of glycosyltransferases (ST3Gal1, ST3Gal2, and ST3Gal4) responsible for the synthesis of α2,3- and α2,6-sialylated glycotopes on the cell surface [134]. The reduction of sialylation of Caco-2 cells significantly inhibited the adhesion of enteropathogenic *E. coli* (EPEC) [23,34]. Straubinger and coworkers [135] showed that in vitro human milk oligosaccharides induce differential expression of glycan-related genes in Caco-2 cells, in contrast to synthetic galactooligosaccharides (GOS), currently added to infant formula to mimic HMOs.

### 5.4. Sialylated Oligosaccharides of Human Milk as Immunomodulators

During inflammatory processes E and P-selectin on the apical surface of endothelial cells recognize and interact with sialyl-Lewis X (sLeX) glycotopes, a part of cell-surface glycoconjugates on leukocytes, which are essential for leukocyte extravasation and mucosal infiltration [98,136]. Similarly, some human milk oligosaccharides might be recognized by immune receptors and in this way modulate the adhesion of cells in breastfed infants [35,83,136,137]. The first experimental data suggesting that HMOs interfere with leukocyte rolling and potentially reduce leukocyte extravasation has been reported by Rudloff and co-workers [77]. Based on previous findings [77,138,139,140], Bode and Jantscher-Krenn [35] speculate that 3′-sialyl-3-fucosyllactose (3′-S,3-FL) present in human milk and more complex HMOs with more than one sLeX glycotope, which allows multivalent binding to selectins, can participate in reduction of leukocyte rolling and adhesion. Moreover, in comparison to sLeX and 3′-S,3-FL alone, the pool of all sialylated HMOs was more effective. In contrast, there was no effect on leukocyte rolling and adhesion when the simplest sialylated trisaccharides (3′-SL and 6′-SL) were used [35,141].

Experimental work of Bode and coworkers [138] demonstrated that sialylated HMOs reduce in vitro the ability of rolling and adhesion of leukocytes, isolated from human peripheral blood, to TNF-alpha-activated human umbilical vein endothelial cells. Additionally, a decrease of monocyte adhesion to human umbilical vein endothelial cells ranging from 24.0% to 52.8% for physiological levels of sialylated fraction of HMOs (0.0125–0.125 mg/L) was observed [138]. Moreover, sialylated HMOs have the ability to ex vivo reduce formation of platelet-neutrophil complexes, which can be more easily activated than circulating neutrophils [36,142]. In this way, the sialylated HMOs can mute immunological processes, such as capacity for phagocytosis and production of the reactive form of oxygen, which are the first steps in the development of necrotizing enterocolitis (NEC) [36,98,143,144]. Of the many sialylated oligosaccharides, an important role in reducing the frequency of occurrence of inflammatory diseases, including NEC, in breastfed infants compared to formula-fed infants is played by DS-LNT, which is one of the most abundant disialylated human milk oligosaccharides and may be used as an anti-inflammatory agent [136,145]. Recently, it was reported that DS-LNT concentrations were lower in breast milk from mothers with preterm infants who developed NEC [145,146]. In light of the above, the authors [98,136,145,146] suggested that estimation of mother’s milk DS-LNT deficiency might be helpful in identifying the increased risk for NEC development. Moreover, enzymatically sialylated galacto-oligosaccharides (Sia-GOS) together with 2′-fucosyllactose might reduce NEC in neonatal rats [145]. Additionally, HMOs added to infant formula in amounts comparable to breast milk induce limited short-term effects on the immature gut in preterm pigs [147]. Additionally, sialylated HMOs through binding to cells may prevent apoptosis [56]. However, as pointed out by authors, the results obtained in animal model, warrant further studies to investigate the underlying mechanisms and to assess safety and efficacy in human neonates.

Milk oligosaccharides affect mucosal immunity and bacterial colonization of breastfed neonates. In a rodent model, it was reported that 3′-SL directly modulates mucosal immunity by induction of inflammation through Toll-like receptor 4 (TLR4) signaling [148]. Additionally, HMOs and BMOs have the ability to modulate the transcriptional response of colonic epithelial cells (HT-29), namely they increase the level of expression of cell surface receptors, chemokines, and an epithelial cell-derived cytokine (IL-17C) and in this way may increase the protection of neonates, while also contributing to the maturation of the intestinal immune response [149]. Moreover, in a mouse model, human milk oligosaccharides such as 2′-fucosyllactose and 6′-sialyllactose reduce the symptoms of food allergy and may be considered as therapeutics in allergic disease [150].

## 6. Sialic Acid in Infant Formulas

An important aspect of newborn and infant feeding is the content of sialic acid in artificial milk. Formula-fed infants only obtain approximately 25% or less of the sialic acid delivered during exclusively breastfeeding; namely mature human milk contains 0.7 g/L of sialic acid, while formula milk based on mature bovine milk contains 0–0.2 g/L [32,110]. Moreover, a majority of sialic acid (70%) present in formula milk is linked with glycoproteins, whereas in human milk, it is linked with HMOs (73%) [32]. However, the absolute concentrations are quite different; namely in human milk the concentrations of sialylated HMOs and bovine milk oligosaccharides (BMOs) are 2–3 g/L and 0.07–0.08 g/L, respectively (Table 2). Moreover, oligosaccharides of human and bovine milk due to differences in concentration and composition show quite different protection against human pathogens [38,47,151,152].

The comparison of N-glycome of human and bovine milk glycoproteins using high performance liquid chromatography and tandem mass spectrometry showed that 12 out of the 38 detected human milk N-glycans were sialylated, while for bovine milk N-glycans 22 out of 51 were sialylated (Table 2) [43]. Another important aspect is the presence of N-glycolyl-5-neuraminic acid (Neu5Gc) in bovine milk and thus in bovine-based formula milk, which does not naturally occur in human milk [32,59,153]. Humans are genetically deprived of the ability to synthesize Neu5Gc due to the lack of an active monooxygenase (exon deletions and/or mutations causing a frameshift in the gene *CMAH*) [15,19,154,155]. Neu5Gc is a derivative of sialic acid, which is formed by hydroxylation of the N-acetyl group at C5 of Neu5Ac [15,19,154,155].

Neu5Gc is classified as a xeno-autoantigen, which can be delivered with red meat and bovine milk or can be synthesized as the end product of abnormal changes of fatty acids [153,156,157]. Moreover, Neu5Gc is not discriminated from Neu5Ac by biochemical metabolic pathways, which can lead to the incorporation of Neu5Gc to newly synthesized glycoconjugates [154]. Glycans decorated with Neu5Gc present on the cell surface may induce the synthesis of anti-Neu5Gc antibodies (called Hanganutziu-Deicher antibodies). The formation of such antigen-antibody complexes might provoke a chronic inflammatory process, which can potentially trigger carcinogenesis [154]. The content of Neu5Gc in bovine-based formula milk is about ~3–5% of the total sialic acid, however, further research is needed to investigate the impact of formula milk-derived Neu5Gc on the health and development of newborns and infants [158,159].

## 7. Sialic Acid in Milk from Milk Bank

Human milk in the intensive care unit for preterm newborns is used not only as a nutrient, but also as the first and the best natural medicine [160]. In some cases when the mother’s own milk is not available, milk deposited by donors in a milk bank can be used. However, the milk collected in a milk bank must be processed by methods, which do not affect the biologically active milk components but are effective in the neutralization of pathogens, to be safe for newborns. A small body of work has examined the effects of pasteurization on the macronutrient and immune-protective protein concentration in donor milk [161,162,163]. To date, only two reports, by Meredith-Dennis and coworkers [162] and Daniels and coworkers [164], have analyzed the impact of pasteurization on HMOs content in banked milk. Use of the Holder pasteurization technique to process donor milk for storage is more favorable for biologically active HMOs containing fucose and sialic acid than retort sterilization [162]. However, this issue requires further investigation.

## 8. Mimicking Human Milk

Milk produced by no other mammal species has similar quantity and diversity of oligosaccharides and glycoconjugates compared to human milk [34,38,165]. However, when breastfeeding is not possible, as a substitute of human milk, formula milk mixtures are used. On the market, two main types of formula milk—mixtures based on bovine milk and mixtures with modified composition of proteins (isolated and hydrolyzed bovine milk proteins)—are available. The majority of human milk oligosaccharides and glycoconjugates such as glycoproteins and glycolipids, showing a protective effect, are not found in bovine milk or protein isolates [59]. For this reason, plant-derived or synthetic prebiotic mixtures of fructooligosaccharides (oligomers of fructose built from two to more than 60 fructose unit and glucose at the reducing end) (FOS) and galactooligosaccharides (oligomers of galactose built from 3 to 10 galactose unit and glucose at the reducing end) (GOS) (the ratio of GOS:FOS is 9:1), which mimic the prebiotic properties of HMOs and support the development of beneficial intestinal microflora, can be added [165,166]. However, GOS and FOS, in contrast to HMOs, do not contain fucose and sialic acid residues, which can be recognized and bound by lectin receptors of sialic acid and fucose-dependent pathogens [34]. Nevertheless, despite the structural differences between HMOs and oligosaccharides such as GOS and FOS, it was reported that formula milk with addition of a mixture of GOS and FOS reduces the occurrence of atopic dermatitis [167], allergic manifestations and infections [168], and also influences the microbiota composition in the infant’s feces and provides softer stools [169].

Another solution to narrowing the compositional gap between human milk and formula milk is the supplementation of infant formula with commercially available free oligosaccharides [56,170]. Thus far, Weichert and coworkers [170] have showed that biotechnologically synthesized oligosaccharides such as 2′-FL and 3-FL effectively inhibit in vitro the adhesion of fucose-dependent pathogens, thus reducing the risk of infection. However, due to the huge technical challenge to synthesize oligosaccharide structures identical to those in human milk, so far only relatively simple structures of oligosaccharides have been obtained on an industrial scale in this way [171]. The latest research by Guo and coworkers [172] reported the method for enzymatic synthesis of a dominant sialylated HMO, namely 6′-SL. It is a milestone, especially considering that traditional chemical synthesis of nine-carbon sialic acids is rather complicated and requires multiple protection and deprotection steps to achieve a specific glycosidic bond [172]. The research currently carried out worldwide paves the way for large-scale synthesis of fucosylated and sialylated oligosaccharides, whose production in the future will make a significant contribution to the food and pharmaceutical industries.

An alternative method of sourcing oligosaccharides is their extraction from animal milk, which can be potentially used in medical and functional foods [173]. Theoretically, another option to obtain HMOs is their isolation from human milk. However, it seems more reasonable to provide the “whole” milk of donor mothers than only “one” fraction isolated.

## 9. Conclusions

Mother’s milk not only has nutritional value but also provides a wide range of biologically active components, which are adapted to meet the needs of newborns and infants. Glycoconjugates and free oligosaccharides unique for human milk are classified as an essential element of innate immunity delivered by the mother, which modulate and enhance the immature newborns.

Studies of recent years indicate that, similar to fucosylated, the sialylated free oligosaccharides as well as glycans attached to proteins and lipids are a key component which, acting locally in the intestine, protects infants from infections and has an impact on the shaping of the intestinal microbiome. Additionally, sialylated oligosaccharides modulate the immunological system of newborns at a cellular level, and are crucial for brain development and probably for newborn and infant growth. Moreover, some human milk sialylated structures are considered as natural prophylactic or therapeutic biomolecules, particularly for preterm newborns. However, it should be pointed out that most of the conclusions on the immune effects are based on in vitro studies, many of which use adult cells.

In light of the above, human milk has a beneficial impact and an advantage over infant formula, which do not provide adequate immunological protection for the developing infants. For the sake of the healthy development of future generations and as a pressing global health goal, feeding newborns with their own mother’s milk or with milk from donor mothers as a gold standard should be supported.

## Figures and Tables

**Table 1:** Sialylated glycoconjugates and oligosaccharides of human milk that may inhibit pathogen and toxin adhesion to human epithelial cells.

Glycotope and glycoconjugate	Pathogen	References
**Human milk oligosaccharides (HMOs)**		
Oligosaccharides with Neu5Acα2,3Galβ1,4	S-fimbriated *Escherichia coli*	[102]
6′-Sialyllactose 3′-Sialyllactose 6′-Sialyl-N-acetyllactosamine	Influenza virus	[103]
3′-Sialyllactose	Human respiratory syncytial virus (RSV) *Helicobacter pylori* *Escherichia coli*	[99,104,105]
Sialylated human milk oligosaccharides	Enterotoxigenic and uropathogenic *Escherichia coli*	[106,107]
**GLYCOPROTEINS**		
Sialylated glycans of human skim milk mucins and S-IgA	S-fimbriated *Escherichia coli*	[108]
Sialylated glycans of human milk glycoproteins	*Helicobacter pylori*	[109]
Sialylated glycans of κ-casein	*Streptococcus mutans*	[110]
Sialylated glycans of lactadherin and mucins in the milk fat globule	Rotavirus	[111]
**GLYCOLIPIDS**		
GM1	Enterotoxigenic *Escherichia coli* (ETEC), Heat labile-toxin of *Escherichia coli* (LT), Cholera toxin of *Vibrio cholerae* (CT)	[70,112,113]
GM1, GM2	Vacuolating cytotoxin A of *Helicobacter pylori* (VacA)	[70,114]
GM2	Human respiratory syncytial virus (RSV)	[70,115]
GM3	Enterotoxigenic *Escherichia coli* (ETEC)	[113]
GM1, GM3, GD3	*Campylobacter jejuni,* *Listeria monocytogenes,* *Salmonella enterica* (Typhi) *Shigella sonnei,* *Helicobacter pylori*	[70,116]
GD3 Neu5Ac(α2,8)Neu5Ac	Enterotoxigenic *Escherichia coli* (ETEC)	[70,112]
Gb_3_	*Shigella dysenteriae* Shiga toxin (Stx)	[65,70,113]
Glycolipids with glycotope: Neu5Acα2,3Gal, Neu5Acα2,6Gal	Enterovirus 71 (EV71)	[70,117]

GM—Monosialoganglioside; GD—Disialoganglioside; Gb—Globotriaosylceramide.

**Table 2:** Comparison of human and bovine milk glycoproteins and free oligosaccharides.

Glycans/Oligosaccharides	Milk Glycoproteins	
	Human [43]	Bovine [43]
Fucosylated N-glycans	24 out of 38 detected	21 out of 51 detected
Sialylated N-glycans	12 out of 38 detected	22 out of 51 detected
N-glycans with Neu5Gc	not detected	9 out of 51 detected
	**Oligosaccharides**	
	**HMOs**	**BMOs**
Total concentration	5–15/20 g/L [34,98]	0.05–0.01 g/L [34,42,98]
Identified structures	>200 [38] >150 [136]	~40 [34,70] 20 [151]
Nonfucosylated neutral	42–55% [56]	14% [151]
Fucosylated oligosaccharides	35–50% [56] 60–80% [47] (~7–16 g/L) *	~1% [34,151] (~0.0005 g/L) *
Sialylated oligosaccharides	12–14% [56] 10–15% [47] (2–3 g/L) *	70% [34,38] 84% [151] (0.035-0.042 g/L) *
Presence of Neu5Gc (xeno-autoantigen)	not detected	5% [38] 2% [151]

* calculated from available data for both lower and higher values of the range. HMOs—Human Milk Oligosaccharides, BMOs—Bovine Milk Oligosaccharides.

## Abbreviations

2′-/3-FL	2′-/3-fucosyllactose
3’-/6’-SL	3’-/6’-sialyllactose
3’-S,3-FL 3’	sialyl-3-fucosyllactose
6’-SLN	6’-sialyllactosamine
AGP	α1-acid glycoprotein
BMOs	Bovine milk oligosaccharides
DS-LNT	Disialyllacto-N-tetraose
FN	Fibronectin
FOS	Fructooligosaccharides
Fuc	Fucose
Gal	Galactose
GalNAc	N-Acetylgalactosamine
GD	Disialoganglioside
Gb	Globotriaosylceramide
GM	Monosialoganglioside
GOS	Galactooligosaccharides
HMOs	Human milk oligosaccharides
IgG	Immunoglobulin G
LBSA	Lipid-bound sialic acid
LF	Lactoferrin
LNFP-I	Lacto-N-fucopentaose I
LNnT	Lacto-N-neotetraose
LST	Lacto-N-tetraoses
NEC	Necrotizing enterocolitis
Neu5Ac	N-Acetylneuraminic acid (sialic acid)
Neu5Gc	N-glycolyl-5-neuraminic acid
RSV	Respiratory syncytial virus
Sia-GOS	sialylated galacto-oligosaccharides
S-IgA	Secretory immunoglobulin A
sLeX	sialyl-Lewis X
